# Supplementary material for: The CryoEM structure of the Saccharomyces cerevisiae ribosome maturation factor Rea1
Source: eLife. 2018 Nov 26;7:e39163. doi: 10.7554/eLife.39163 (PMC6286127; doi:10.7554/eLife.39163)
Supplement: Supplementary file 1. — Data collection and refinement statistics for the presented cryoEM maps and structural models. VPP = volta phase plate. [file elife-39163-supp1.docx]

|  | Rea1 AAA+ ring  WT ADP | Rea1 linker  WT ADP | Rea1  WT AMPPNP | Rea1  ΔAAA2L-H2α  AMPPNP |
| --- | --- | --- | --- | --- |
| Data collection | | | | |
| Voltage | 300kV | | | |
| Target defocus | 1.8-3.4 | 1.8-3.4 | 1.8-3.4 | 0.5-0.6 (VPP) |
| Dose [e^-^/Å^2^] | 46.2 | 46.2 | 50 | 49.6 |
| Micrographs | 23230 | 23230 | 6797 | 2479 |
| Particles in the final refinement | 76071 | 432556 | 55442 | 20724 |
| Refinement | | | | |
| Resolution (Å) | 4.4 | 3.9 | 4.3 | 7.8 |
| Map sharpening B-factor (Å^2^) | -180 | -180 | -118.03 | -258.3 |
| Average B factor (Å^2^) | 133.2 | 35.7 | 84.9 | 82.1 |
| Root mean square deviations (RMSD’s) | | | | |
| Bond lengths (Å) | 0.002 | 0.005 |  |  |
| Bond angles (°) | 0.61 | 0.79 |  |  |
| Ramachandran plot | | | | |
| Favored (%) | 92.75 | 95.58 |  |  |
| Allowed (%) | 7.25 | 4.26 |  |  |
| Outliers (%) | 0 | 0.16 |  |  |
| Molprobity | | | | |
| Clash score | 1.68 | 2.7 |  |  |
| Rotamer outliers (%) | 0 | 0.1 |  |  |
| Overall score | 1.38 | 1.37 |  |  |
